# Optimized production of a biologically active *Clostridium perfringens* glycosyl hydrolase phage endolysin PlyCP41 in plants using virus-based systemic expression

**DOI:** 10.1186/s12896-019-0594-7

**Published:** 2019-12-21

**Authors:** Rosemarie W. Hammond, Steven M. Swift, Juli A. Foster-Frey, Natalia Y. Kovalskaya, David M. Donovan

**Affiliations:** 1 USDA ARS NEA BARC Molecular Plant Pathology Laboratory, Beltsville, MD 20705 USA; 2 USDA ARS NEA BARC Animal Biosciences and Biotechnology Laboratory, Beltsville, MD 20705 USA; 3 Oak Ridge Institute for Science and Education, ORISE, Beltsville, MD 20705 USA

**Keywords:** Alternative antimicrobial, Bacteriophage, Endolysin, *Nicotiana benthamiana*, Plant production of recombinant proteins, Plant virus-based gene expression, *Clostridium perfringens*, Potato virus X

## Abstract

**Background:**

*Clostridium perfringens*, a gram-positive, anaerobic, rod-shaped bacterium, is the third leading cause of human foodborne bacterial disease and a cause of necrotic enteritis in poultry. It is controlled using antibiotics, widespread use of which may lead to development of drug-resistant bacteria. Bacteriophage-encoded endolysins that degrade peptidoglycans in the bacterial cell wall are potential replacements for antibiotics. Phage endolysins have been identified that exhibit antibacterial activities against several Clostridium strains.

**Results:**

An *Escherichia coli* codon-optimized gene encoding the glycosyl hydrolase endolysin (*PlyCP41*) containing a polyhistidine tag was expressed in *E. coli*. In addition, The *E. coli* optimized endolysin gene was engineered for expression in plants (*PlyCP41p*) and a plant codon-optimized gene (*PlyCP41pc*), both containing a polyhistidine tag, were expressed in *Nicotiana benthamiana* plants using a potato virus X (PVX)-based transient expression vector. PlyCP41p accumulated to ~ 1% total soluble protein (100μg/gm f. wt. leaf tissue) without any obvious toxic effects on plant cells, and both the purified protein and plant sap containing the protein lysed *C. perfringens* strain Cp39 in a plate lysis assay. Optimal systemic expression of PlyCP41p was achieved at 2 weeks-post-infection. PlyCP41pc did not accumulate to higher levels than PlyCP41p in infected tissue.

**Conclusion:**

We demonstrated that functionally active bacteriophage PlyCP41 endolysin can be produced in systemically infected plant tissue with potential for use of crude plant sap as an effective antimicrobial agent against *C. perfringens*.

## Background

*Clostridium perfringens* is a Gram-positive, rod-shaped, spore-forming, anaerobic bacterium that is commonly found in the environment and is present in the intestines of animals and humans. The bacterium produces four major toxins and is the third leading cause of human foodborne illnesses; outbreaks are frequently associated with exposure to raw meat or poultry which has not been maintained properly [1, 2]. *C. perfringens* also causes gas gangrene in humans that have been subjected to severe injuries. In wild and domestic animals, it causes enteric diseases. In poultry, *C. perfringens* causes necrotic enteritis, characterized by necrotic lesions on the intestinal mucosa, which can be very costly to the poultry industry [3]. Control of clostridia in commercial poultry has commonly been by the feeding of sub-therapeutic amounts of antibiotics added to animal feed [4, 5], however concern that antibiotic resistance may develop from the continual use of antibiotics has led to reduced or banned use of antibiotics in some countries, resulting in increased cases of necrotic enteritis in poultry [6, 7]. Therefore, there is increasing interest in the development of alternative and specific antimicrobials to control *C. perfringens* and other bacterial animal pathogens.

Bacteriophage lysins are highly evolved, phage-encoded enzymes that hydrolyze peptidoglycans, the major structural component of bacterial cell walls. Bacteriophage and their derived lysins have been explored as tools to control bacterial infections [8–15]. Several bacteriophages of *C. perfringens* have been characterized [16] and putative phage endolysins have been identified to control *C. perfringens* [17]. Two recombinant, native lysins produced in and purified from *Escherichia coli*, Ply26F and Ply39O, lysed their parental *C. perfringens* host strains in addition to other strains of Clostridium, but did not lyse other bacterial species [18]. The demonstrated modular nature of endolysins [11, 19] led Swift et al. to design a thermally stable endolysin, a chimeric protein composed of the catalytic domain derived from an endolysin of the thermophilic bacteriophage, ɸGVE2, fused to a cell wall binding domain derived from an endolysin of *C. perfringens* bacteriophage ɸCP26F. The resulting protein, PlyGVE2CpCWB, was active over a range of pH and salt conditions and was more resistant to elevated temperatures, demonstrating the ability to impart new properties to these catalytic enzymes [20].

To identify new lysins against *C. perfringens*, the genomes of 43 *C. perfringens* strains were searched for prophage regions predicted to encode endolysins. Sequence analysis and annotation resulted in the identification of a glycosyl hydrolase endolysin from the source strain Cp41, with the resulting endolysin designated PlyCP41 [21]. Bacterially-produced recombinant PlyCP41 lysed 75 strains of *C. perfringens*, which included isolates from chickens, pigs, and cows [21].

Plant production of antimicrobials is advantageous because of lower production costs, smaller risks of pathogen contamination, the ability to produce a large amount of protein, and their simplicity to produce and deliver in feed. Bacteriophage endolysins have been synthesized in stably transformed tobacco chloroplasts [22, 23], however this method is laborious and requires time and selection to identify transgene inheritance. Alternatively, plant virus-based transient expression, in which the inserted mRNA encoding a recombinant protein is replicated by the plant virus, can produce high levels of protein within a short period. For that reason, virus-based expression is an attractive alternative to transformation and has been used to produce many recombinant proteins, including antimicrobials and bacteriophage endolysins in *Nicotiana benthamiana* [24–28].

The purpose of this study was to produce PlyCP41 in plants (PlyCP41p) and to examine the activity of the purified protein and lysin-containing crude plant sap against *C. perfringens*. In our study, we compared the production of PlyCP41 in bacteria and plants and found that PlyCP41 was present in soluble bacterial fractions, eliminating the need for laborious re-solubilization and refolding steps required when recombinant proteins form inclusion bodies, leading to poor recoveries of active protein. PlyCp41p and the plant codon-optimized PlyCP41pc were expressed in plant sap at 1% total soluble protein in *N. benthamiana* leaves using a potato virus X (PVX)-based vector. PlyCP41 expressed both in bacteria and in plant tissues lysed *C. perfringens* in a plate lysis assay. In the future, phage lysins produced in plants could be added as lysates or dried plant tissue to animal feeds for reducing the bacterial colonization of the poultry gut to improve animal health and food safety.

## Results

### Expression and purification of recombinant PlyCP41 in bacteria

**Fig. 1:**
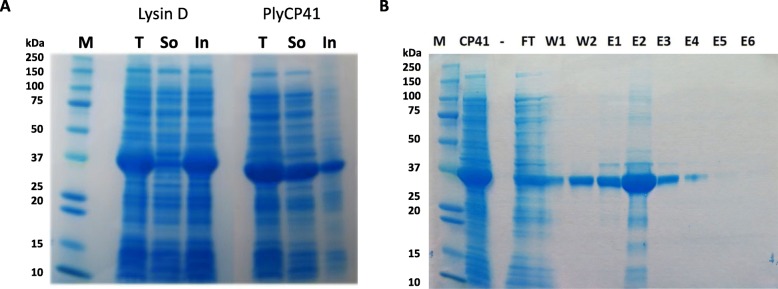
**a** Protein production in *E. coli*. Bacterial cultures containing pET21a: Lysin D or pET21a: PlyCP41 (PlyCP41) were induced by addition of IPTG and proteins were purified using the BugBuster reagent. Total (T), soluble (So), and inclusion body (IB) fractions were collected. Five μl aliquots were run on a protein gel. **b** Purification with PlyCP41 with Ni-NTA columns under native conditions. CP41, Soluble fraction from BugBuster fraction added to the Ni-NTA column; FT, flow through; W1, Wash 1; W2, Wash 2; E1, Elute 1; E2, Elute 2, E3, Elute 3; E4, Elute 4; E5, Elute 5; E6, Elute 6. Both gels were stained with SimplyBlue Safe Stain. M = Precision Plus Kaleidoscope protein standards

An expression construct encoding an *E. coli*-codon optimized gene was used to produce a histidine-tagged PlyCP41 in *E. coli* strain BL21(DE3). Analysis of protein fractions by SDS-PAGE following IPTG-induction revealed that PlyCP41 (335 amino acids, 38.5 kDa) was predominantly localized in the soluble fraction compared to a Lysin D (361 amino acids, 40.8 kDa) that was localized predominantly in the insoluble inclusion bodies (Fig. 1a). PlyCP41 was easily purified to almost complete homogeneity from the soluble fraction using a Ni-NTA resin under native conditions (Fig. 1b). The concentration of PlyCP41 in the second elution fraction was ~ 2 mg/mL. PlyCP41 also eluted in the wash buffers, resulting in a concentration of 100–200 μg/mL, and may have resulted from a wash stringency that was too high.

### Expression of recombinant PlyCP41 in plant tissues

**Fig. 2:**
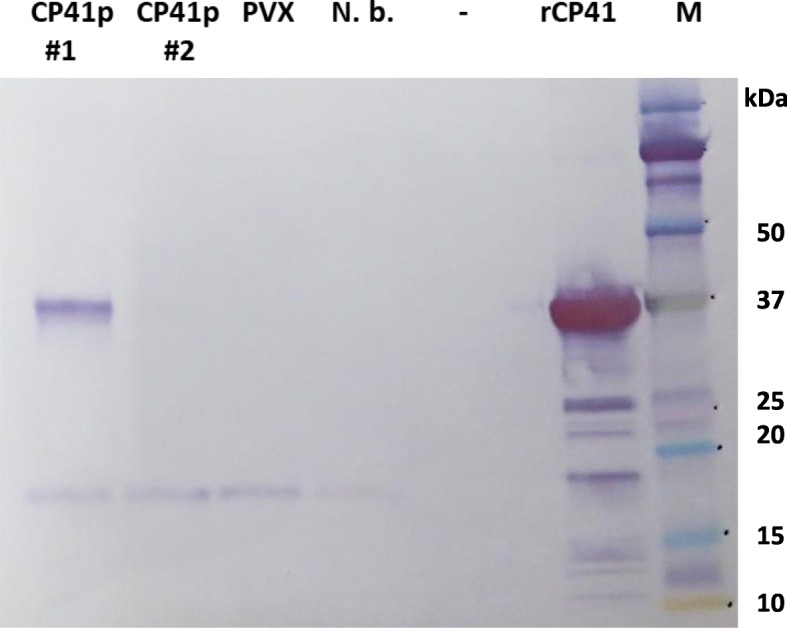
PlyCP41p production in leaf tissue collected from virus-infected plants. Western blot using the Penta-His antibody of plant extracts from leaf discs collected 9 days’ post-infiltration CP41p (plant 1, symptomatic leaf), CP41p (plant 2, asymptomatic leaf), PVX, N.b. (healthy plant). rCP41 = 2 μg of purified recombinant PlyCP41 from bacteria. M = Precision Plus Kaleidoscope protein standards

For plant expression, the *PlyCP41* gene was engineered into a PVX-plant virus expression vector, pGDPVXMCS. In the resulting construct, pGDPVXMCS: PlyCP41p, plant gene expression is under control of the *Cauliflower mosaic virus* 35S transcriptional promoter. Co-agroinfiltration of *A. tumefaciens* strain EHA105 containing pGDPVXMCS: PlyCP41p and pGDp19 into *N. benthamiana* leaves led to systemic PVX virus infection and synthesis of PlyCP41p protein in upper, systemically infected, symptomatic leaves at 9 days’ post-infiltration (Fig. 2; CP41p#1), while there was no detectable PlyCP41p protein in an asymptomatic leaf of the same plant (Fig. 2; CP41p#2). pGDp19 encodes a plant virus-derived silencing suppressor protein that facilitates high levels of gene expression from the PVX construct [29]. There was no obvious phenotypic difference between plants infected with PVX and those infected with PVX containing the lysin insert (not shown).

### Lytic activity of bacterial and plant-produced lysins

**Fig. 3:**
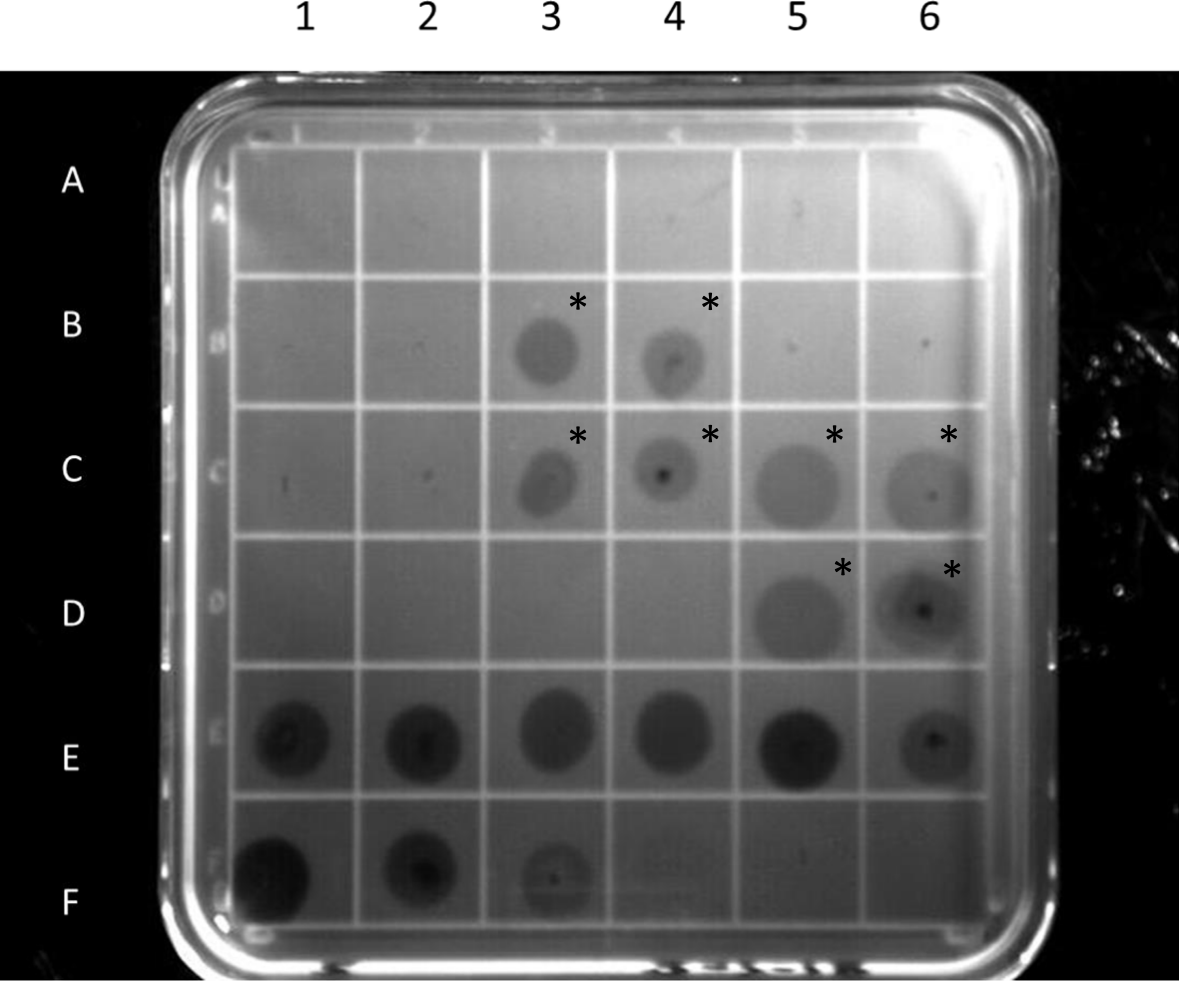
Plate lysis assay of protein samples on *Clostridium perfringens* Cp39 confluent plates. Spot assays were conducted as described in the Materials and Methods and a photographic image of the plate was taken 30 min after application of the samples. The contents of the wells are as follows: A1-A6, Negative control, His-purified fractions from *E. coli*: BL21 pET21a; B1- B4- plant virus samples in PBS buffer; B1 & B2-empty PVX virus; B3 & B4-PVX virus with PlyCP41p; B5-PBS buffer; B6-elution buffer control; C1-C4- plant virus samples in “10:90” buffer (50 mM NaH_2_PO_4_ pH 7.0, 30 mM NaCl, 25 mM imidazole, 3% glycerol). C1&C2-empty PVX; C3&C4-PVX with PlyCP41p; C5–10 μL PVX with PlyCP41p in PBS buffer; C6–10 μL PVX with PlyCP41p in PBS buffer; D1-D4- empty; D5–10 μL PVX with PlyCP41p in “10:90 buffer”; D6–10 μL PVX with PlyCP41p in “10:90 buffer” E1-E6- *E. coli* PlyCP41 fractions purified on Ni-NTA column (FT, W1, W2, E1, E2, E3); F1- F4–10 μg, 1 μg, 0.1 μg, 0.001 μg of PlyCP41; F5 10:90 buffer. Asterisks indicate the plant extracts containing the PlyCP41p protein

To ensure that the lysins were active against *C. perfringens* bacteria, a plate lysis assay was performed (Fig. 3). PlyCP41 fractions purified from bacteria (shown in Fig. 1b, E1–6) were all efficient in lysing the bacteria when compared to control, purified lysin (Fig. 3, F1–3). Crude sap extracts obtained from plants expressing PlyCP41p were also active against the bacteria (Fig. 3, B3–4, C3–6, D5–6, indicated by asterisks on the figure) while extracts from healthy plants and PVX-infected plants were not (Fig. 3, B1–2, C1–2). This assay revealed that clearing occurred within 30 min at room temperature after addition of the samples. The plant extracts (spot equivalent to 2 mg of fresh weight (f. wt.) tissue and 200 ng of purified PlyCP41p) had similar clearing compared to 0.1 μg of purified, *E. coli*-produced PlyCP41 (Fig. 3, compare B-3 to F-3).

### Solubility and optimization of the production capacity in PlyCP41p-expressing plants

**Fig. 4:**
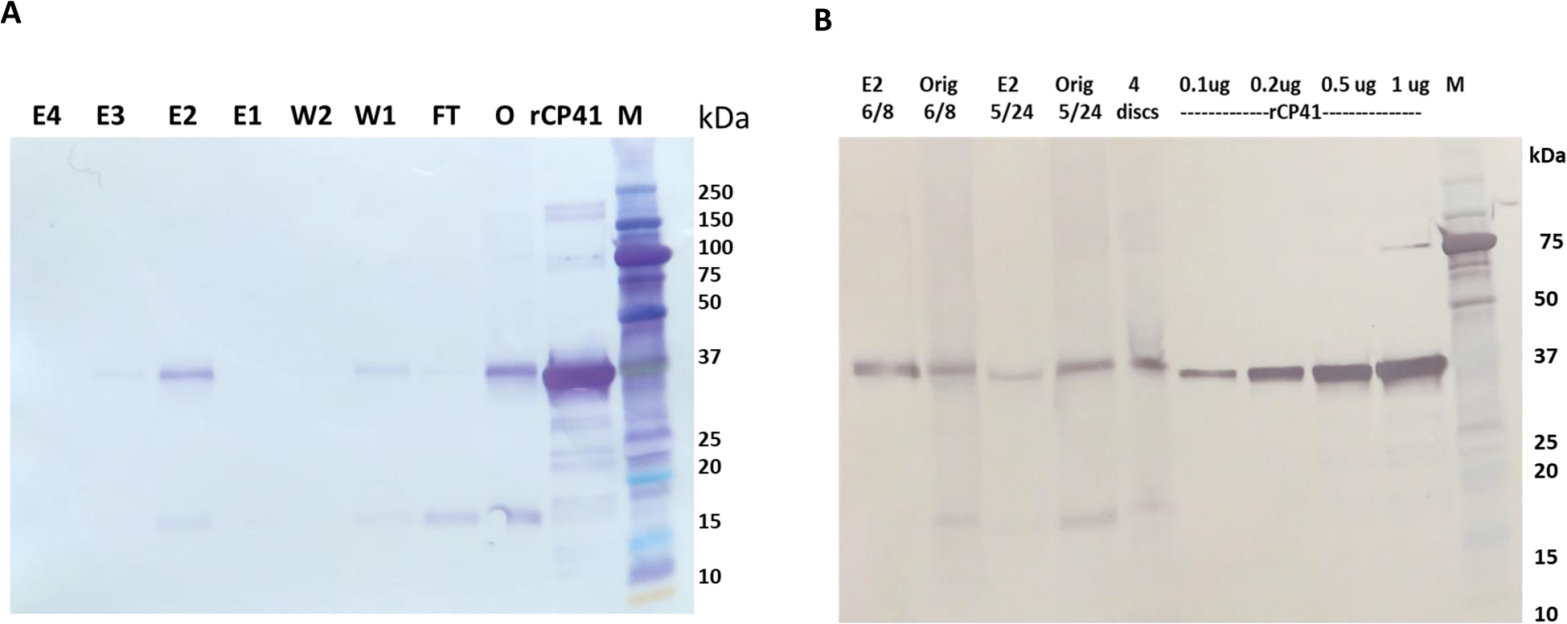
Purification and quantitation of plant-expressed PlyCP41p. **a** Plant extracts were processed using Ni-NTA columns under native conditions and analyzed by Western blot of protein using the Penta-His antibody. O, leaf sample extract from virus-infected plants were added to the Ni-NTA column; FT, flow through; W1, Wash 1; W2, Wash 2; E1, Elute 1; E2, Elute 2, E3, Elute 3; E4, Elute 4. rCP41 = 2 μg of Ni-NTA purified PlyCP41 from bacteria. M = Precision Plus Kaleidoscope protein standards. **b** Quantitation of PlyCP41p in plant extracts. Lanes containing Ni-NTA-purified PlyCP41 from bacteria-concentrations are 0.1 μg, 0.2 μg. 0.5 μg, and 1 μg. The unpurified plant extract (Orig) and the Elution 2 fraction (E2) from Ni-NTA purifications (6/8) and (5/24). The 6/8 purification included protease inhibitor in the extraction buffer. 1 μl of a 100 μl extraction of 4 leaf discs (20 mg plant sample) M = Precision Plus Kaleidoscope protein standards

For purification of PlyCP41p to homogeneity from crude plant sap using the Ni-NTA resin, the same native buffer conditions used to purify the bacterially-expressed lysin, described in Materials and Methods, were applied. PlyCP41p was easily recovered in the second elution fraction under native conditions (Fig. 4a), identical to what was observed for PlyCP41 from bacteria (Fig. 1b), suggesting that PlyCP41p is also soluble in plant tissues, thus facilitating its ease of purification for future studies. To quantify the amounts of PlyCP41p that could be recovered plant extracts, we determined that from 100 mg of leaf protein, we could purify 100 μg of PlyCP41p in elution fraction 2 at 200 ng/ul, or 1 g/1 kg f.wt. tissue (Fig. 4b, lanes E2).

**Fig. 5:**
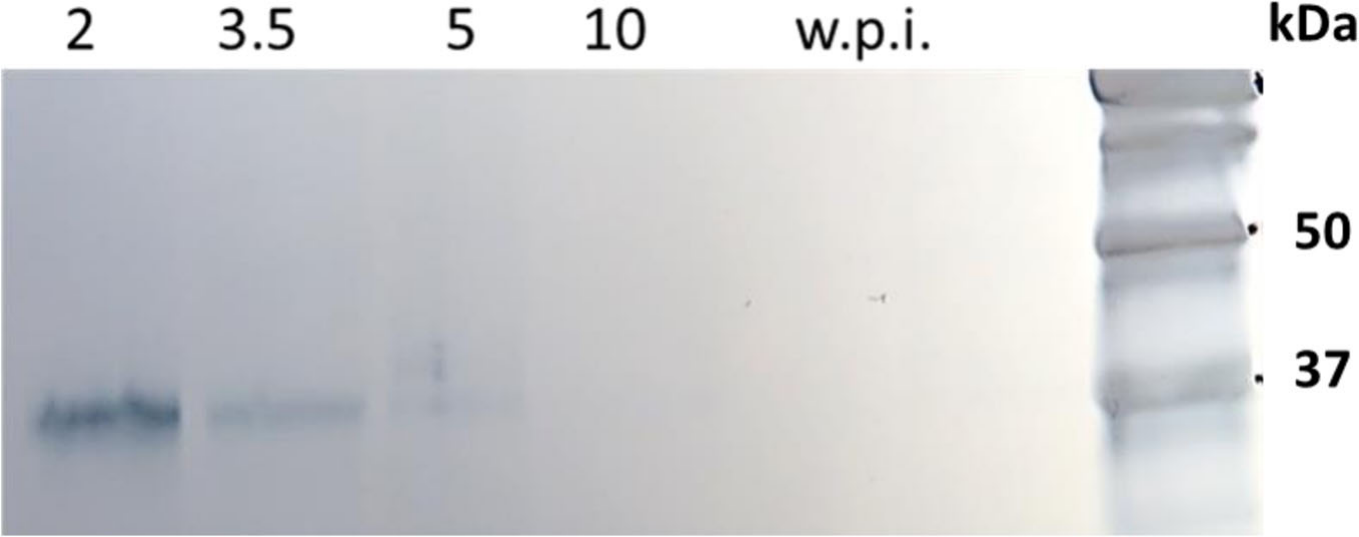
PlyCP41p protein expression in *N. benthamiana* plants post-infiltration. Leaf disc samples were collected from infiltrated plants at 2, 3.5, 5, and 10 weeks’ post-infiltration. Western blot analysis was performed using the Penta-His conjugate. N.b., healthy *N. benthamiana*. M = Precision Plus Kaleidoscope protein standards. This Western blot shows that plant tissue needs to be harvested within 2 weeks’ post-inoculation (w.p.i) to achieve the highest amount of PlyCP41p protein production

**Fig. 6:**
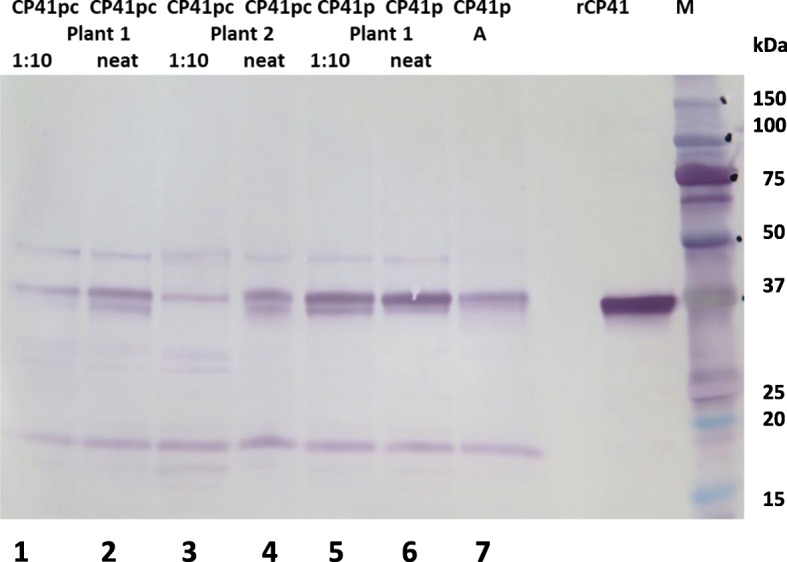
Comparison of the expression of PlyCp41p and PlyCp41pc in plants. Western blot of plant tissue collected 13 days post-infiltration. Neat (O.D. 600 nm = 2.7) and 1:10 (O. D at 600 nm = .27) represent dilutions of Agrobacterium cultures used to infiltrate plants with pGDPVXMCS: PlyCP41pc (CP41pc) or pGDPVXMCS:PlyCP41p mixed in a 1:10 dilution with Agrobacterium containing pGDp19. CP41p (A) designates a plant that was mechanically inoculated from a plant 22 days’ post-infiltration. This sample represents 7 days’ post-infection. rCP41 = 2 μg. M = Precision Plus Kaleidoscope protein standards

Expression of PlyCP41p was substantially greater at 2 weeks’ post-inoculation of the leaf compared to 3.5 weeks, and expression was further reduced at later time points (Fig. 5). This suggests that the optimal time to harvest infected leaf tissue to achieve the highest recovery of PlyCP41p was within 2 weeks’ post-inoculation (Fig. 5). Although the leaf tissue was positive for the PVX virus as assayed using the immunostrips, and PVX titer appeared to be equivalent to leaves in which the protein was expressed, protein expression in older plants was reduced. When older, infected leaf tissue was used to mechanically inoculate healthy *N. benthamiana* plants (Fig. 6, lane 7) indicating that the virus retained the lysin insert and can express the lysin protein when passaged.

To achieve the highest production of PlyCP41 protein in plants using the PVX-expression vector, we engineered a plant codon-optimized lysin gene (PlyCP41pc) into the same PVX vector and compared its accumulation to that of *E. coli* codon-optimized gene (PlyCP41p) in plants 13 days’ post-infiltration. (Fig. 6). PlyCP41pc (lanes 1–4) did not accumulate to higher levels than PlyCP41p (lanes 5, 6), however we did find that using undiluted Agrobacterium cultures (neat, lanes 2, 4, 6) led to higher accumulation of the recombinant proteins in systemically infected leaf tissue than a 1:10 dilution (Fig. 6, lanes 1, 3, 5).

## Discussion

In this study, we report the first successful expression of an active PlyCP41, a previously characterized phage-derived endolysin that targets *C. perfringens* [21] in systemically infected *N. benthamiana* plants using a PVX-based expression vector (Fig. 2). Cytoplasmic expression of the PlyCP41p gene in *N. benthamiana* led to expression levels of 1% TSP/ (0.1 mg/gm fresh weight plant tissue). In addition, we constructed and expressed a plant codon-optimized PlyCP41pc gene in plants and equivalent amounts of PlyCP41pc protein were produced. We demonstrated that for optimal PlyCP41p protein production, plant material needed to be harvested within 2 weeks’ post-inoculation (Fig. 5) whether the initial inoculation occurred via agroinfiltration or inoculation of sap from virus-infected plants to healthy plants (Fig. 5). Notably, there were no obvious additional local or systemic symptoms in plants infected with PVX expressing the lysin compared with those infected with PVX alone (data not shown), allowing recovery of the recombinant protein 2–3 weeks’ post-inoculation. Although plants were infected with PVX for several weeks, the recombinant protein levels decreased even though the lysin gene was stable in the virus, as evidenced by the ability of sap extracts from these plants to generate new infections from which high levels of lysin were produced (Fig. 5). We have observed this phenomenon over several experiments and have found that we can reliably use sap from initially infected plants to scale up protein production in subsequently inoculated plants. This observation might be explained by the plants RNA silencing response in virus-infected plants [29, 30], however we do not have experimental evidence to support that theory.

In the prokaryotic expression system that we utilized to produce the positive control, PlyCP41 expressed from the *E. coli*-optimized gene was predominantly located in the soluble fraction of lysed bacterial cells (Fig. 1b), in contrast to a similarly expressed endolysin (Lysin D) which was predominantly localized in the inclusion fraction. Solubility facilitated ease of purification using native conditions and nickel resin from the bacteria and may have facilitated ease of purification using native conditions from plant sap (Fig. 1b and Fig. 4a). There are numerous computational tools that predict protein solubility and aggregation as increasing solubility for industrial and therapeutic applications is of great value [31, 32]. Future examination of the protein sequence of the soluble PlyCP41 and other endolysins that were insoluble using these tools may aid in the design of future endolysins with improved solubility without impact on activity.

Substitution of codons encoding the same amino acid can affect the expression of proteins when attempting to express proteins in different hosts. Codon optimization of PlyCP41 for expression in plants, PlyCP41pc, did not result in increased levels of recombinant protein production (Fig. 6) in contrast to our earlier studies where poor expression in plants of an *E. coli*-optimized triple fusion protein, composed of phage lysin cassettes, was improved by plant codon optimization and allowed accumulation of the protein to 0.12 mg/gm f. wt. tissue [26]. Although the impact of codon optimization on heterologous gene expression is unpredictable, there are several cases in the literature where 2 to 3-fold increases have been reported [33].

The plate lysis assay indicated that both the bacterial- and plant-expressed lysins possessed lytic activity against *C. perfringens* strain Cp39 (Fig. 3). Incubation of clarified plant sap and purified protein produced clear zones within 30 min of application to the bacterially-embedded agar revealing that plant components in the sap did not inhibit the lytic activity of PlyCP41.

Kazanaviĉiūtė et al. [28] recently reported the production of six biologically-active phage lysins in plants, using a deconstructed, transient tobacco mosaic virus-based vector expression system, by infiltration or spraying of leaves with the Agrobacterium constructs. Tissue was collected 5–6 days’ post infiltration from the ‘inoculated’ leaves, and the lysins were active against a panel of *C. perfringens* serotypes. The authors estimated lysin production at 30% total soluble protein (TSP) based on visual inspection of stained gels, and purification from 150 to 1150 μg/gm f. wt. depending upon the lysin. Although they could produce up to 10-fold higher amounts of lysin in the infiltrated leaves, their system does not result in systemic virus infection. The advantage of our expression system is that we have production of the recombinant protein systemically and can passage the virus to young plants with resulting increased production and scale-up.

Our results suggest that crude plant lysates, or unprocessed plant tissue, containing recombinant phage lysins could be effective additives to animal feeds to control bacterial infections to improve animal health and food safety. Limitations of oral delivery, such as stability in the poultry gastrointestinal tract and effective concentration in the gut, will be addressed in follow-up in vitro and in vivo studies. Although we expressed PlyCP41p in *N. benthamiana*, expression of PlyCP41p in alternative plant species is being explored and will be used to generate the lysins using additional plant virus-based vectors.

## Conclusions

Using a plant virus-based systemic expression system we produced, within two-week post-inoculation of plants, a biologically active phage endolysin with demonstrated activity against *C. perfringens.* As the lysin was active in unpurified, crude plant lysates, purification of the protein is not required. The lysates or dried plant tissue could be added to animal feeds for reducing *C. perfringens* colonization of the poultry gut to improve animal health and food safety and reduce production costs for the industry. This technology could be applied for the expression of other bacteriophage-derived endolysins for use as alternative antimicrobials for control of animal diseases.

## Methods

### Plasmid constructions

The PlyCP41 gene (GenBank KX884995) was synthesized as an *E. coli* codon optimized construct and cloned into pET21a (Novagen®, Millipore Sigma, Billerica, MA) by GenScript (Piscataway, NJ) [21]. PlyCP41 contains a C-terminal 6xHis-tag to facilitate purification using an Ni-NTA resin. pET21a: PlyCP41 was transformed and maintained in *E. coli* TOP10 cells (Life Technologies, Carlsbad, CA). The plasmid was transformed into *E. coli* strain BL21 (DE3) (Stratagene, La Jolla, CA) for protein production. As a control for expression in bacteria, plasmid pET21a: Lysin D (unpublished), which encodes another bacteriophage endolysin of similar size, was also transformed in BL21 cells for protein production only in *E. coli.* A plant codon-optimized CP41 gene containing a C-terminal 6xHis-tag (PlyCP41pc) with a CAI index of 0.92 was synthesized by Genscript USA (Piscataway, NJ) and was cloned in the pJET1.2 vector [33] (Additional file 1: Figure S1).

For expression in plants, the PlyCP41 coding region was amplified from the pET21a vector using primer pair BKEYS08 (5′- CC*GGATCC*AACAATGCTGAAGGGTATCGACGTTAGC-3′) and BKEYS09 (5′- CC*AAGCTT*TCAGTGGTGGTGGTGGTGGTGCTCGAG-3′) and AmpliTaq DNA polymerase (Applied Biosystems, Foster City, CA) and the amplicon was cloned in the pCR4 vector (Life Technologies) for sequence analysis. The gene was then isolated by restriction digestion using *Bam*HI and *Hin*dIII and inserted into a similarly digested intermediate pSKAS vector that is based on pBluescript SK+ and containing nt 4945 to nt 6541 of the pP2C2S PVX-based vector [34] and an expanded multiple cloning site [35]. The intermediate pSKAS vector allows engineering of insertions into a smaller plasmid vector from which the insertion can be transferred into the full-length virus-based vector. The resulting pSKAS: CP41 plasmid was digested with *Apa*I and *Spe*I and the insert was isolated and cloned into the similarly digested pGDPVXMCS plasmid (containing the full-length, PVX genome [36]), creating pGDPVXMCS: CP41p. The *E. coli* codon-optimized gene was maintained in this construct.

The plant codon-optimized PlyCP41pc gene was amplified from pJET: CP41p using oligonucleotide primers BKEYS17 (5′- C*CCATGG*AACAATGCTTAAGGGAATTGATGTTTCTGAAC-3′) and BKEYS18 (5- CC*GAATTC*CTAATGATGATGATGATGATGAAGTTTC 3′). The resulting amplicon was cloned into pCR4 vector. The PlyCP41pc gene was isolated from pCR4:CP41p by digestion with *Nco*I and *Eco*RI and cloned into the *Nco*I/*Eco*RI sites of the pSKAS vector, creating pSKAS: PlyCP41pc. Digestion of this plasmid with *Apa*I/*Spe*I released a fragment that was ligated into *Apa*I/*Spe*I digested pGDPVXMCS, creating pGDPVXMCS:PlyCP41pc. For all cloning, PCR products and gene fragments were gel purified from 1% agarose/TBE gels using the QIAquick Gel Extraction Kit (Qiagen GmbH, Hilden, Germany), vectors and inserts were ligated using T4 DNA ligase (New England Biolabs), and transformed into competent Top 10 *E. coli* cells (Life Technologies). The plasmid constructs were maintained the *E. coli* TOP 10 cells using appropriate antibiotics and plasmid DNAs were purified using the QIAprep Miniprep kit (Qiagen GmbH). All plasmids were sequenced for verification (Genscript USA).

### Bacterial protein overexpression and purification using the BugBuster reagent

The pET21A:PlyCP41 construct was transformed into *E. coli* BL21 (DE3) cells for protein induction. Briefly, 5 mL of LB broth was inoculated with a loop of bacterial cells harboring the construct. After overnight incubation in a shaking incubator at 37 °C, 100 μl of cells was inoculated into 2 mL of LB and the culture was grown for 2 h. An aliquot of the culture was removed as a non-induced control. For protein induction, isopropyl-β-D-1-thiogalactopyranoside (IPTG) was then added to the cell cultures at a final concentration of 2 mM and the cultures were incubated with shaking at 37 °C for a further 2 h, during which time aliquots were removed at after 1 and 2 h for analysis by SDS-PAGE. For large scale protein purification of induced proteins, 500 μl of an overnight culture was added to 50 mL of LB in a 125 ml Erlenmeyer flask. Bacterial pellets were recovered from 50 mL of IPTG-induced bacterial cultures grown at 37 °C by centrifugation at 4000 x g for 20 min in a Jouan CR422 centrifuge (Saint-Herblain, France) as previously described [24]. The BugBuster Master Mix Protein Extraction Reagent (Novagen, Madison, WI) and protease inhibitor cocktail for plant cells) (Sigma Chemical Co.) (1 μL cocktail per 100 μl of BugBuster Reagent) was added to the bacterial pellet to prepare the bacterial lysates and extract total proteins. The extraction was carried out per manufacturer’s instructions to obtain total, soluble, and inclusion body fractions. For determination of protein concentrations, the Bradford assay using the Quick Start™ Bradford 1xDye Reagent and Quick Start™Bovine Serum Albumin (BSA) Standard Set (Bio-Rad Laboratories, Hercules, CA) were used per manufacturer’s instructions.

### Agroinfiltration of *N. benthamiana* leaves

*Agrobacterium tumefaciens* strain EHA105 was transformed with the pGDPVXMCS, pGDPVXMCS: PlyCP41p, and pGDPVXMCS:PlyCP41pc plasmids and the bacteria were plated on Luria Broth-glucose (LBg) agar containing rifampicin and kanamycin at 50 μg/mL each. Colonies which appeared after incubation of the plates at 28 °C were inoculated into 5 mL of liquid LBg broth and grown overnight at 28 °C and 250 rpm in a shaking incubator. The cultures were centrifuged for 10 min at 4000 x g at 25 °C in a Jouan CR422 centrifuge. The bacterial pellets were gently resuspended in 2 mL of infiltration medium (10 mM MES, 10 mM MgCl_2,_ pH 5.7) and 4 μL of 1 M acetosyringone (Sigma Chemical Co.) was added. After incubation at ambient room temperature for 4 h, the cultures were individually mixed with a culture of similarly prepared EHA105 containing the plasmid pGDp19 (encoding a plant viral-encoded suppressor protein [29]) at a ratio of 1:10 (pGDp19: pGDPVXMCS construct). Three to four young leaves *N. benthamiana* plants at the 5–6 leaf stage were infiltrated on the abaxial side of the leaf using a needleless syringe. Plants were grown in the laboratory at 27 °C and were observed for symptom production and monitored for virus infection using PVX AgriStrips following manufacturer’s instructions (Eurofins BioDiagnostics, Inc., Longmont, CO).

### CP41 extraction from *N. benthamiana* plants

Four leaf discs (~ 20 mg tissue) were collected and placed into an eppendorf tube to test for PlyCP41p and PlyCP41pc protein production. The leaf samples were ground in 100 μl of the CellLytic™ P Plant Cell Lysis/Extraction Reagent (Sigma Chemical Co., Saint Louis, MO) containing 1 μl of plant protease inhibitor cocktail (Sigma Chemical Co.) using a blue pestle. Cell debris was removed by centrifugation at 4 °C, and the supernatant was combined with an equal volume of Laemmli buffer (BioRad Laboratories, Hercules, CA). After boiling for 10 mins, an aliquot of the sample was applied to a 10–20% Tris-glycine gel as described below.

### Protein gel electrophoresis and Western blot analysis

Proteins were resolved by SDS-PAGE analysis on a Novex 10–20% Tris-glycine gradient mini gels (Life Technologies) under denaturing conditions using manufacturer’s instructions. The proteins were visualized by staining with SimplyBlue Safe Stain (Life Technologies). Alternatively, the proteins were transferred to a 0.45 μM nitrocellulose membrane (Life Technologies). The membranes were subsequently incubated with a 1:1000 dilution of Anti-His HRP Conjugate solution (Penta His HRP Conjugate Kit (Qiagen) following manufacturer’s instructions followed by development using the TMP Membrane Peroxidase Substrate System (Kirkegaard and Perry, Gaithersburg, MD) to visualize the proteins.

### Protein purification using nickel resin (IMAC) under native conditions

To purify bacterial and plant expressed His-tagged proteins under native conditions, we used the Ni-NTA His-Bind Resins Kit and the Ni-NTA Buffer kit (Novagen) following manufacturer’s instructions to purify PlyCP41 from the soluble fraction obtained previously from *E. coli* using the Bug Buster reagent (above). The plant PlyCP41p protein was also purified from plant sap using the Ni-NTA His-Bind Resin under native conditions. Plant tissue was ground in a chilled mortar and pestle using Binding Buffer (BB; 50 mM NaH_2_PO_4_, pH 8.0; 300 mM NaCl; 10 mM imidazole) from the His-Bind Resins Kit, to which the plant protease inhibitor cocktail (Sigma Chemical Co.) was added in a ratio of 1 μl cocktail to 100 μl BB. The Bradford assay described above was used to determine protein concentrations in the fractions.

### Testing the lytic activity of expressed proteins against *C. perfringens*

The plate lysis (spot) assay was performed essentially as described previously [20]. *C. perfringens* strain Cp39 cultures were propagated to mid-log phase (OD600 = 0.4–0.6) in 50 mL BHIB, where upon the cells were centrifuged at 5000 g for 30 min. The cell pellet was washed with 50 mL lysin buffer (50 mM NH_4_OAc, 10 mM CaCl_2_, 1 mM DTT, pH 6.2) and pelleted again. The cells were suspended in 1.0 mL lysin buffer. Ten milliliters of 50 °C semisolid BYC ss agar (37 g/L brain heart infusion powder, 5 g/L yeast extract, 0.5 g/L cysteine, 7 g/L Bacto agar) was added to the cells and then the cells were poured into a sterile 6 × 6 grid square petri dish. The plates sat 20 min at room temperature to solidify the agar. Ten μL of the purified endolysin or plant sap was then spotted onto the plate and allowed to air dry 20 min. The purified lysins were in diluted in “10:90” buffer (50 mM NaH_2_PO4, pH 7, 30 mM NaCl, 2 mM imidazole, 3% glycerol). Plant sap was prepared from uninfected tissue, leaf tissue from plants infiltrated with the empty pGDPVXMCS, PVX-based vector, and plants infiltrated with the pGDPVXMCS: PlyCP41p plasmid by grinding 4 leaf discs (~ 20 mg) with a pestle in 100 μL 1 x PBS buffer (Bio-Rad, Hercules, CA) in an Eppendorf tube. After one round of centrifugation at 16,000 x g for 5 min, the supernatant was removed from the pellet containing cellular debris and 10 μL of plant sap was applied to the plate as described. A positive lytic reaction was determined by visible clearing of the turbidity of the bacterial cells. The plate was observed for development of visible clearing and then incubated overnight in an anaerobic chamber at 37 °C.

## Supplementary information

**Additional file 1: Figure S1.** Plant codon-optimized PlyCP41pc gene. A. Nucleotide sequence of the PlyCP41pc gene and explanatory notes and encoded protein. B. Alignment of *E. coli* optimized gene PlyCP41 (and identical PlyCP41p) nucleotide sequence (lower line in black) and plant codon-optimized gene PlyCP41pc (upper line in red). Yellow boxes indicate the modified sequences in PlyCP41pc.

## Data Availability

All the data and materials presented in the article are available from the corresponding author upon reasonable request.

## Abbreviations

CAI: Codon adaptation index
His: Histidine
PVX: Potato virus X
TSP: Total soluble proteins

## References

[1] Olsen SJ, MacKinon LC, Goulding JS, Bean NH, Slutsker L (2000). Surveillance for foodborne-disease outbreaks- United States, 1993-1997. Morb Mortal Wkly Rep.

[2] Scallan E, Hoekstra RM, Angulo FJ, Tauxe RV, Widdowson MA, Roy SL, Jones JL, Griffin PM (2011). Food-borne illness acquired in the United States—major pathogens. Emerg Infect Dis.

[3] McDevitt RM, Brooker JD, Acamovic T, Sparks NHC (2006). Necrotic enteritis; a continuing challenge for the poultry industry. Worlds Poult Sci J.

[4] Devriese LA, Daube G, Hommez J, Haesebrouck F (1993). In vitro susceptibility of *Clostridium perfringens* isolated from farm animals to growth-enhancing antibiotics. J Appl Bacteriol.

[5] Watkins KL, Shryock TR, Dearth RN, Saif YM (1997). In vitro antimicrobial susceptibility of *Clostridium perfringens* from commercial Turkey and broiler chicken origin. Vet Microbiol.

[6] Casewell M, Friis C, Marco E, McMullin P, Phillips I (2013). The European ban on growth-promoting antibiotics and emerging consequences for human and animal health. J Antimicrob Chemother.

[7] Van Immerseel F, De Buck J, Pasmans F, Huyghebaert G, Haesebrouck F, Ducatelle R (2004). *Clostridium endolysins* in poultry: an emerging threat for animal and public health. Avian Pathol.

[8] Fischetti VA (2011). Exploiting what phage have evolved to control gram-positive pathogens. Bacteriophage.

[9] Dong H, Zhu C, Chen J, Ye X, Huang Y-P (2015). Antibacterial activity of *Stenotrophomonas maltophilia* endolysin P28 against both gram-positive and gram-negative bacteria. Front Microbiol.

[10] Nakonieczna A, Cooper C, Gryko R (2015). Bacteriophages and bacteriophage-derived endolysins as potential therapeutics to combat gram-positive spore forming bacteria. J Appl Microbiol.

[11] Schmelcher M, Donovan DM, Loessner MJ (2012). Bacteriophage endolysins as novel antimicrobials. Future Microbiol.

[12] Schmelcher M, Loessner MJ (2016). Bacteriophage endolysins: applications for food safety. Curr Opin Biotechnol.

[13] Miller RW, Skinner J, Sulakvelidze A, Mathis GF, Hofacre CL (2010). Bacteriophage therapy for control of necrotic enteritis of broiler chickens experimentally infected with *Clostridium perfringens*. Avian Dis Dig.

[14] Becker SC, Roach DR, Chauhan VS, Shen Y, Foster-Frey J, Powell AM (2016). Triple-acting lytic enzyme treatment of drug-resistant and intracellular *Staphylococcus aureus*. Sci Rep.

[15] Gervasi T, Horn N, Wegmann U, Dugo G, Narbad A, Mayer MJ (2014). Expression and delivery of an endolysin to combat *Clostridium perfringens*. Appl Microbiol Biotechnol.

[16] S. Bruce, V. Nikolay, B. Brian, A. Cesar, K. Johnna, Simmons Mustafa, A. Edward, R. Gregory (2012). Bacteriophages of Clostridium perfringens. Bacteriophages.

[17] Seal BS (2013). Characterization of bacteriophages virulent for *Clostridium perfringens* and identification of phage lytic enzymes as alternatives to antibiotics for potential control of the bacterium. Poult Sci.

[18] Simmons M, Donovan DM, Siragusa GR, Seal BS (2010). Recombinant expression of two bacteriophage proteins that lyse *Clostridium perfringens* and share identical sequences in the C-terminal cell wall binding domain of the molecules but are dissimilar in their N-terminal active domains. J Agric Food Chem.

[19] Oliveira H, Melo LD, Santos SB, Nobrega FL, Ferreira EC, Cerca N (2013). Molecular aspects and comparative genomics of bacteriophage endolysins. J Virol.

[20] Swift S, Seal B, Garrish J, Oakley B, Hiett K, Yeh H, Donovan D (2015). A Thermophilic phage endolysin fusion to a *Clostridium perfringens*-specific cell wall binding domain creates an anti-Clostridium antimicrobial with improved thermostability. Viruses.

[21] Swift S, Waters JJ, Rowley DT, Oakley BB, Donovan DM (2018). Characterization of two glycosyl hydrolases, putative prophage endolysins, that target *Clostridium perfringens*. FEMS Microbiol Lett.

[22] Oey M, Lohse M, Kreikemeyer B, Bock R (2009). Exhaustion of the chloroplast protein synthesis capacity by massive expression of a highly stable protein antibiotic. Plant J.

[23] Stoffels L, Taunt HN, Charalambous B, Purton S (2017). Synthesis of bacteriophage lytic proteins against *Streptococcus pneumoniae* in the chloroplast of *Chlamydomonas reinhardtii*. Plant Biotechnol J.

[24] Kovalskaya N, Hammond RW (2009). Expression and functional characterization of the plant antimicrobial snakin-1 and defensin recombinant proteins. Protein Expr Purif.

[25] Kovalskaya N, Foster-Frey J, Donovan DM, Bauchan G, Hammond RW (2016). Antimicrobial activity of bacteriophage endolysin produced in *Nicotiana benthamiana* plants. J Microbiol Biotechnol.

[26] Kovalskaya NY, Herndon EE, Foster-Frey JA, Donovan DM, Hammond RW (2019). Antimicrobial activity of bacteriophage derived triple fusion protein against *Staphylococcus aureus*. AIMS Microbiol.

[27] Starkevič U, Bortesi L, Virgailis M, Ružauskas M, Giritch A, Ražanskienė A (2015). High-yield production of a functional bacteriophage lysin with antipneumococcal activity using a plant virus-based expression system. J Biotechnol.

[28] Kazanaviĉiūtė V, Misiūnas A, Gleba Y, Giritch A, Ražanskienė A (2018). Plant-expressed bacteriophage lysins control pathogenic strains of *Clostridium perfringens*. Sci Rep.

[29] Qiu W, Park JW, Scholthof HB (2002). Tombusvirus P19-mediated suppression of virus-induced gene silencing is controlled by genetic and dosage features that influence pathogenicity. Mol Plant-Microbe Interact.

[30] Ghoshal B, Sanfaçon H (2015). Symptom recovery in virus-infected plants: revisiting the role of RNA silencing mechanisms. Virology.

[31] Broom A, Jacobi Z, Trainor K, Meiering EM (2017). Computational tools help improve protein stability but with a solubility tradeoff. J Biol Chem.

[32] Chan P, Curtis RA, Warwicker J (2013). Soluble expression of proteins correlates with a lack of positively-charged surface. Sci Rep.

[33] Webster GR, Teh AY, Ma JK (2016). Synthetic gene design-the rationale for codon optimization and implications for molecular pharming in plants. Biotechnol Bioeng.

[34] Chapman S, Kavanagh TA, Baulcombe DC (1992). Potato virus X as a vector for gene expression in plants. Plant J.

[35] Kovalskaya N, Zhao Y, Hammond RW (2011). Antibacterial and antifungal activity of a snakin-defensin hybrid protein expressed in tobacco and potato plants. Open Plant Sci J.

[36] Lim H-S, Vaira AM, Domier LL, Le SC, Kim HG, Hammond J (2010). Efficiency of VIGS and gene expression in a novel bipartite potexvirus vector delivery system as a function of strength of TGB1 silencing suppression. Virology.

