# Supplementary material for: Optimized production of a biologically active Clostridium perfringens glycosyl hydrolase phage endolysin PlyCP41 in plants using virus-based systemic expression
Source: BMC Biotechnol. 2019 Dec 21;19:101. doi: 10.1186/s12896-019-0594-7 (PMC6925876; doi:10.1186/s12896-019-0594-7)
Supplement: Supplementary file 1 — Additional file 1: Figure S1. Plant codon-optimized PlyCP41pc gene. A. Nucleotide sequence of the PlyCP41pc gene and explanatory notes and encoded protein. B. Alignment of E. coli optimized gene PlyCP41 (and identical PlyCP41p) nucleotide sequence (lower line in black) and plant codon-optimized gene PlyCP41pc (upper line in red). Yellow boxes indicate the modified sequences in PlyCP41pc. [file 12896_2019_594_MOESM1_ESM.docx]

**Figure S1.** Plant codon-optimized PlyCP41pc gene. A. Nucleotide sequence of the PlyCP41pc gene and explanatory notes and encoded protein. B. Alignment of *E. coli* optimized gene PlyCP41 (and identical PlyCP41p) nucleotide sequence (lower line in black) and plant codon-optimized gene PlyCP41pc (upper line in red). Yellow boxes indicate the modified sequences in PlyCP41pc.

A. Plant codon-optimized CP41 gene:

GGGCC*Caac*ATGCTTAAGGGAATTGATGTTTCTGAACATCAAGGTAGAATTGATTGGGAGAGGGTTAAAGGAAACATCGATTTCGCTATCCTTAGAGCTGGATACGGTAGGAATAACATCGATAAGCAATTCATTAGAAACATTGAAGAGTGTAATAGGTTGTGCATTCCAGTTGGAATCTATTGGTTTTCATACGCTTGGAATGAGGAGATGGCTAAGAATGAGGCTAGATATGTTCTTGAGGCTATTAAAGGTTATAGGGTTGATTACCCTATCTCTTACGATCTTGAGTACGATACTTTGAACTACGCTTCTAAGAATGGTGTTACTATTGGAAAGAGATTGGCTACAGATATGGTTAAAGCTTTCTGTGATGAGATTAATAGAAATGGTTACAGGGCTATGAATTACACTAACCAAGATTTTCTTTTGAATAAGTTCTACATGAATGAGCTTACAAACTACCCATTGTGGTACGCTTGGTACAACTCTAAATTGAACAGAGATTGCGCTATCTGGCAATATTCTGAATCAGGACAAGTTCCTGGTATTGGAGGTGCTTCAGTTGATATGAACTACTGTTACGAAGATTTTCTTAAGAAAGATTTCACTTTGGAGAACGCTACTACATGCAACGTTGATACAGAACTTAACATTAGGGCTAAGGGAACTACAGGTGCTACTATTGTTGGATCTATTCCAGCTGGAGATAGGTTTAGAATTAAGTGGGTTGATTCAGATTATTTGGGATGGTATTACATTGAGTACCAAGGAATTACAGGTTATGTTTCTCAAGATTACGTTGAAAAGCTTCAAATGGCTACTACATGTAACGTTGATTCAGTTTTGAACGTTAGGGCTGAGGGAAACACTTCTTCAAACATCGTTGCTACAATTAATCCTGGTGAAGTTTTTAGAATCGATTGGGTTGATTCTGATTTCATCGGTTGGTACAGGATCACTACAGCTAATGGTGCTAATGGTTTCGTTAAATCAGATTTCGTTAAGAAACTTCATCATCATCATCATCATTAGACTAGT

Double-underline: restriction sites used for initial design of gene. Primers containing different restriction sites were used to move the gene into the plant virus-based expression vector plasmid through a 2-step process and intermediate plasmid.

Italics: surrounding plant translational consensus sequence (We also used this when we amplified the E. coli codon-optimized gene to express in plants)

ATG: start codon

Dotted line: coding for HIS-tag (6 histidine residues)

Wavy line: stop codon

Plant codon-optimized CP41 protein:

MLKGIDVSEHQGRIDWERVKGNIDFAILRAGYGRNNIDKQFIRNIEECNRLCIPVGIYWFSYAWNEEMAKNEARYVLEAIKGYRVDYPISYDLEYDTLNYASKNGVTIGKRLATDMVKAFCDEINRNGYRAMNYTNQDFLLNKFYMNELTNYPLWYAWYNSKLNRDCAIWQYSESGQVPGIGGASVDMNYCYEDFLKKDFTLENATTCNVDTELNIRAKGTTGATIVGSIPAGDRFRIKWVDSDYLGWYYIEYQGITGYVSQDYVEKLQMATTCNVDSVLNVRAEGNTSSNIVATINPGEVFRIDWVDSDFIGWYRITTANGANGFVKSDFVKKLHHHHHH

B. Alignment of PlyCP41pc and PlyCP41 nucleotide sequences

PlyCP41pc ATGCTTAAGGGAATTGATGTTTCTGAACATCAAGGTAGAATTGATTGGGAGAGGGTTAAAGGAAACATCGATTTCGCTATCCTTAG

PlyCP41 ATGCTGAAGGGTATCGACGTTAGCGAGCACCAGGGCCGTATTGATTGGGAACGTGTGAAGGGTAACATCGACTTCGCGATTCTGCG

AGCTGGATACGGTAGGAATAACATCGATAAGCAATTCATTAGAAACATTGAAGAGTGTAATAGGTTGTGCATTCCAGTTGGAATCT

TGCGGGTTACGGCCGTAACAACATCGATAAGCAATTTATCCGTAACATTGAGGAATGCAACCGTCTGTGCATCCCGGTTGGTATTT

ATTGGTTTTCATACGCTTGGAATGAGGAGATGGCTAAGAATGAGGCTAGATATGTTCTTGAGGCTATTAAAGGTTATAGGGTTGAT

ACTGGTTCAGCTATGCGTGGAACGAGGAAATGGCGAAGAACGAGGCGCGTTACGTTCTGGAAGCGATCAAAGGCTACCGTGTGGAC

TACCCTATCTCTTACGATCTTGAGTACGATACTTTGAACTACGCTTCTAAGAATGGTGTTACTATTGGAAAGAGATTGGCTACAGA

TATCCGATTAGCTACGACCTGGAATATGATACCCTGAACTATGCGAGCAAGAACGGTGTTACCATCGGCAAACGTCTGGCGACCGA

TATGGTTAAAGCTTTCTGTGATGAGATTAATAGAAATGGTTACAGGGCTATGAATTACACTAACCAAGATTTTCTTTTGAATAAGT

CATGGTGAAAGCGTTTTGCGATGAAATTAACCGTAACGGTTACCGTGCGATGAACTATACCAACCAGGATTTCCTGCTGAACAAGT

TCTACATGAATGAGCTTACAAACTACCCATTGTGGTACGCTTGGTACAACTCTAAATTGAACAGAGATTGCGCTATCTGGCAATAT

TTTACATGAACGAGCTGACCAACTATCCGCTGTGGTACGCGTGGTATAACAGCAAACTGAACCGTGATTGCGCGATCTGGCAGTAC

TCTGAATCAGGACAAGTTCCTGGTATTGGAGGTGCTTCAGTTGATATGAACTACTGTTACGAAGATTTTCTTAAGAAAGATTTCAC

AGCGAGAGCGGTCAAGTTCCGGGCATTGGTGGCGCGAGCGTGGACATGAACTACTGCTATGAGGACTTCCTGAAGAAAGATTTTAC

TTTGGAGAACGCTACTACATGCAACGTTGATACAGAACTTAACATTAGGGCTAAGGGAACTACAGGTGCTACTATTGTTGGATCTA

CCTGGAAAACGCGACCACCTGCAACGTTGATACCGAGCTGAACATCCGTGCGAAGGGTACCACCGGCGCGACCATCGTTGGTAGCA

TTCCAGCTGGAGATAGGTTTAGAATTAAGTGGGTTGATTCAGATTATTTGGGATGGTATTACATTGAGTACCAAGGAATTACAGGT

TTCCGGCGGGCGACCGTTTCCGTATTAAATGGGTGGACAGCGATTACCTGGGTTGGTACTATATCGAATATCAAGGTATTACCGGC

TATGTTTCTCAAGATTACGTTGAAAAGCTTCAAATGGCTACTACATGTAACGTTGATTCAGTTTTGAACGTTAGGGCTGAGGGAAA

TACGTTAGCCAGGATTATGTGGAGAAGCTGCAAATGGCGACCACCTGCAACGTGGACAGCGTTCTGAACGTGCGTGCGGAGGGTAA

CACTTCTTCAAACATCGTTGCTACAATTAATCCTGGTGAAGTTTTTAGAATCGATTGGGTTGATTCTGATTTCATCGGTTGGTACA

CACCAGCAGCAACATCGTGGCGACCATTAACCCGGGCGAGGTTTTCCGTATCGATTGGGTGGACAGCGATTTTATCGGCTGGTACC

GGATCACTACAGCTAATGGTGCTAATGGTTTCGTTAAATCAGATTTCGTTAAGAAACTT------CATCATCATCATCATCATTAG

GTATTACCACCGCGAACGGTGCGAACGGCTTCGTTAAAAGCGACTTTGTGAAGAAACTGCTCGAGCACCACCACCACCACCACTGA
